# Biomarkers of cardiovascular injury and stress are associated with increased frequency of ventricular ectopy: a population-based study

**DOI:** 10.1186/s12872-016-0407-z

**Published:** 2016-11-22

**Authors:** Julia Brox Skranes, Gunnar Einvik, Silje Kjeka Namtvedt, Anna Randby, Harald Hrubos-Strøm, Jon Brynildsen, Tor-Arne Hagve, Virend K. Somers, Helge Røsjø, Torbjørn Omland

**Affiliations:** 1 Division of Medicine, Akershus University Hospital, Lørenskog, Norway; 2 Asker and Bærum Emergency Unit, Sandvika, Norway; 3 Division of Surgery, Akershus University Hospital, Lørenskog, Norge; 4 Division of Diagnostics and Technology, Akershus University Hospital, Lørenskog, Norway; 5 Division of Cardiovascular Diseases, Department of Internal Medicine, Mayo Foundation for Medical Education and Research, Rochester, MN USA; 6 Institute of Clinical Medicine, University of Oslo, Oslo, Norway

**Keywords:** Arrhythmias, NT-proBNP, troponin I, C-reactive protein

## Abstract

**Background:**

Asymptomatic ventricular arrhythmias are common and associated with increased risk of cardiovascular mortality. Cardiac troponins, natriuretic peptides and C–reactive protein (CRP) are also predictive of adverse cardiovascular events in the general population, but limited information is available on the relationship between these biomarkers and ventricular ectopy in a community-based population.

The objectives were to evaluate the associations between ventricular ectopic activity and N-terminal pro-B-type natriuretic peptide (NT-proBNP), high sensitivity-troponin I (hs-TnI) and hs-CRP in a community-based setting.

**Methods:**

We performed a 24 h Holter-recording and blood sampling in 498 subjects. Premature ventricular complexes (PVC) were classified as frequent at >5/h and the presence of any bigeminy, trigeminy or non-sustained ventricular tachycardia were classified as complex ventricular ectopy. The associations between biomarkers and ventricular arrhythmias were investigated by univariate and multivariate logistic regression analyses.

**Results:**

Frequent PVC’s and complex ventricular ectopy were detected in 46 (9%) and 47 (9%) participants respectively, and were associated with significantly (*p* < 0.001) higher concentrations of NT-proBNP and hs-TnI. The association between NT-proBNP and both frequent PVC’s (*p* = 0.020) and complex ventricular ectopy (*p* = 0.001) remained significant after adjusting for conventional risk markers in multivariate analyses.

**Conclusion:**

Increased level of NT-proBNP was independently associated with ventricular ectopy, whereas no independent association was observed between hs-TnI and hs-CRP levels and ventricular ectopy in this community-based sample.

**Electronic supplementary material:**

The online version of this article (doi:10.1186/s12872-016-0407-z) contains supplementary material, which is available to authorized users.

## Background

Cardiovascular (CV) biomarkers play an increasingly important role in the diagnosis of cardiac diseases, including the use of cardiac troponins to diagnose acute myocardial infarction [1] and B-type natriuretic peptide (BNP) and N-terminal (NT)-proBNP to diagnose heart failure (HF) [2]. Recently, high sensitivity (hs) assays for determination of very low levels of circulating troponins have been developed [3], and we and others have demonstrated that hs cardiac troponin I (hs-TnI) and T (hs-TnT) levels are predictive of adverse cardiovascular events in patients with stable coronary artery disease [4, 5], as well as in community-based cohorts [6, 7]. Likewise, BNP and NT-proBNP concentrations are strongly associated with cardiovascular risk in the general population [8] and low-level increments of C-reactive protein (CRP), as measured by high-sensitive assays, also predict risk in patients at low or moderate risk for coronary heart disease (CHD) [9, 10].

Sudden cardiac death, often caused by ventricular arrhythmias, is a common cause of death both in patients with established CHD, as well as in the general population [11–13]. Asymptomatic ventricular arrhythmias are commonly observed in community-based samples [14], and have in some studies been linked to increased risk of sudden cardiac death and death from cardiovascular causes [15, 16]. As the established CV biomarkers are associated with mortality in the general population, these biomarkers may also predict asymptomatic ventricular arrhythmias in the general population. However, currently only sparse data are available concerning the association between CV biomarkers and the frequency of arrhythmias in community-based cohorts [17]. Accordingly, the objective of the current study was to assess the potential associations between the concentrations of hs-TnI, NT-proBNP and hs-CRP and the frequency of asymptomatic ventricular ectopy in a community-based sample.

## Methods

### Study design

**Fig. 1:**
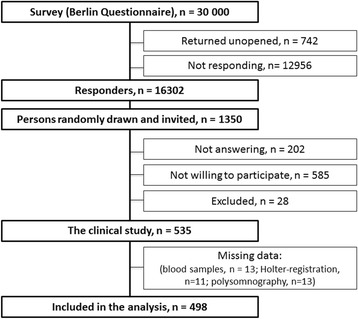
Flow-diagram of the inclusion procedure in Akershus Sleep Apnea Project, and participants in the present substudy

This is a substudy of the Akershus Sleep Apnea Project (ASAP), which is a community-based, cross-sectional study conducted in south Eastern Norway. In a two-phased design, we first randomly drew 30 000 persons between 30 and 65 years of age from the National Population Register (Fig. 1). All participants received the Berlin Questionnaire, a standardized questionnaire for classifying the risk for obstructive sleep apnea (OSA) [18], by mail. Of the 16302 (55.7%) responders, 1772 persons were randomly drawn as a pool for inclusion in phase 2. A 2:1 ratio of high risk:low risk participants according to the Berlin questionnaire in the Phase 2 clinical sample resulted in oversampling of participants with risk factors for obstructive sleep apnea, i.e. snoring, daytime sleepiness, obesity and hypertension. In addition, persons with previous CHD, otitis media surgery or diabetes were oversampled. Predefined age-dependent strata were made to ensure gender equilibrium and a 2:1 ratio of high risk:low risk participants according to the Berlin Questionnaire. Invitations were made by telephone, and 585 of the invited refused to participate, 202 were not reached after three attempts, and 28 persons were excluded. Exclusion criteria of the study included use of continuous positive airway pressure, physical impairment, pregnancy and insufficient Norwegian language skills. In the end, 535 (46.7% of the contacted) persons participated in the clinical phase of the study, while 442 persons from the pool of 1772 were not contacted as the predefined strata were completed. Further details of the recruitment protocol have been described previously [19].

### Data collection

All participants (*n* = 535) arrived at noon and stayed over-night at Akershus University Hospital, Stensby where collection of all of the data was performed.

### Blood sampling and analysis

Fasting blood samples were collected from an antecubital vein in the morning, and were centrifuged within one hour. Serum samples were stored at – 20°C for a maximum of 20 days, before they were transported to the central biobank and frozen at −80°C pending later measurements. Sampling and storing failed for 4 participants. hs-TnI was measured using the ARCHITECT *STAT* hs Troponin assay (Abbott Diagnostics, Illinois, USA) as previously reported [20]. NT-proBNP was measured by electrochemiluminescent immunoassay (Roche Diagnostics, Basel, Switzerland) with a lower detection limit of 5 pg/ml. The hs-CRP measurements were performed using a latex immunoassay kit on a COBAS INTEGRA400 (Roche Diagnostics, Basel, Switzerland), with a lower detection limit of 0.1 pg/mL. Analyses of biomarkers were not performed for 9 participants due to lack of available plasma. Creatinine, glucose and lipids were measured by standard laboratory methods. The creatinine clearance was estimated by the Cockcroft-Gault formula [21].

### Electrocardiography

**Fig. 2:**
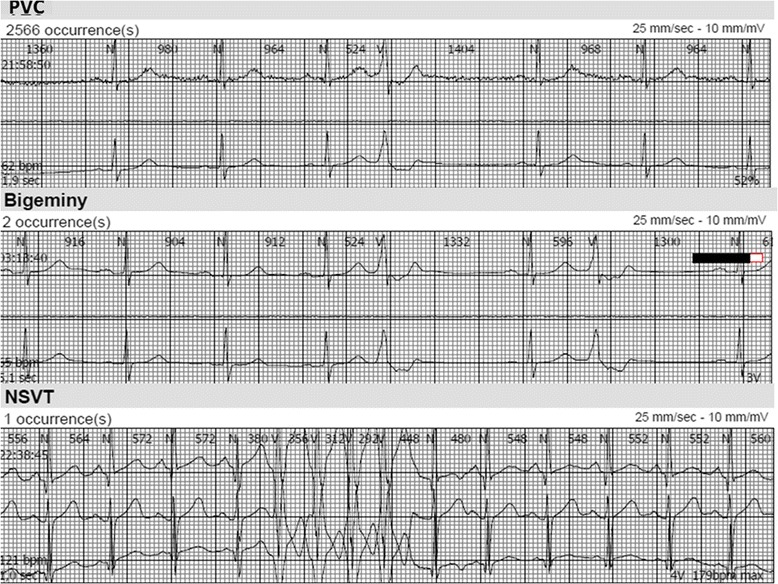
Examples of premature ventricular contraction (PVC) and complex ventricular ectopy

All participants underwent an in-hospital 5-channel ambulatory electrocardiography (Medilog AR12, Oxford Instruments Medical, Surrey, United Kingdom) for assessment of cardiac arrhythmias. Recordings with a shorter duration than 10 h, or with technical failure, were excluded from the analysis (*n* = 10). The mean length of the recordings was 18.3 h. The electrocardiography recordings were automatically analyzed by a software engine (Medilog Darwin, ScanMed Medical, Gloucestershire, United Kingdom) according to predefined criteria. A premature ventricular complex (PVC) was defined as a N-V complex >15% shorter than the previous 3 N-N complexes. All PVC’s were counted and dichotomized at a cut off of 5/h. We defined complex ventricular ectopy as any bigeminy (≥3 consecutive V-N complexes), trigeminy (≥3 consecutive V-N-N complexes), or nonsustained ventricular tachycardia (≥3 consecutive V complexes). One researcher, blinded for the participants’ medical background and biomarker levels, manually reviewed and re-scored all recordings. A second researcher manually reviewed 10% of the recordings. The inter-rater correlation was 0.99. Examples of frequent PVC and complex ventricular ectopy are shown in Fig. 2.

### Demographic variables and cardiovascular risk factors

The participants were asked about history of CHD (myocardial infarction or coronary artery revascularization), history of congestive heart failure, use of antihypertensive medication, diabetes mellitus and current smoking habits and underwent a standard physical examination. Blood pressure was measured in the sitting position after 15 min rest by an automatic device (Dinamap, ProCare 400, GE HealthCare, Milwaukee, WI, USA). The mean of the two last out of three recordings was calculated and used in analysis. The Sokolow-Lyon criteria were used for estimation of left ventricle hypertrophy [22]. Body mass index (weight in kg/ height in meters squared) was calculated. The participants underwent in-hospital polysomnography, as previously reported [19]. Data were manually scored using the Somnologica 3.2 software (Flaga-Medcare, Buffalo, New York), 13 registrations were not analyzed due to technical failure or short sleep duration. The apnea-hypopnea index (AHI) was calculated using the average number of apneas (airflow <10% of reference in more than 10s) plus hypopneas (airflow <30% in more than 10s and subsequent ≥4% fall in oxygen saturation) per hour of sleep.

### Statistical analysis

Due to skewed distribution of data, we transformed NT-proBNP, hs-TnI, CRP and AHI concentrations logarithmically prior to the analysis. Categorical data are presented as absolute numbers and percentages, continuous data as mean (standard deviation) or median (interquartile range). The associations between biomarkers and ventricular arrhythmias were investigated by univariate and multivariate logistic regression analyses. According to a previous report from the same study, OSA may be associated with increased risk of PVC’s [23], consequently we included the AHI as covariate in addition to the conventional cardiovascular risk factors (age, gender, history of CHD, history of diabetes, history of hypertension, body mass index, systolic blood pressure, total cholesterol:HDL cholesterol ratio). Covariates significantly (*p* < 0.05) associated with arrhythmias in univariate analyses were first entered into multivariate models. The cardiovascular biomarkers were subsequently added to make separate multivariate models to evaluate whether hs-TnI, NT-proBNP or hs-CRP added significant incremental information to the regression models. The results are reported as odds ratio (OR) and 95% confidence intervals (CI) and a *p* value (2-sided) < 0.05 was considered statistically significant for all analyses. All statistical analyses were performed with the SPSS statistical software package, version 20 (SPSS Inc., Chicago, USA).

## Results

### Patient characteristics

A total of 498 participants had a complete data set in this substudy. More than 5 PVC/h were observed in 46 participants (9%), while non-sustained ventricular arrhythmia, bigeminy and trigeminy were observed in 13, 26 and 27 participants, respectively. As 19 persons had more than one type of complex ventricular ectopy, a total number of 47 persons (9%) had any complex ventricular ectopy.

Demographic and clinical characteristics according to the presence or absence of more than 5 PVC/h or any complex ventricular arrhythmia are summarized in Tables 1 and 2. No patients reported congestive heart failure.

**Table 1:** Demographic and clinical characteristics in patients with and without > 5 PVC/h

	<5 PVC/h *n* = 452	>5 PVC/h *n* = 46	OR (95% CI)	*P*
Age (mean, SD)	48 (11)	56 (9)	1.09 (1.05–1.12)	<0.001
Male gender (n, %)	243 (54)	29 (63)	1.47 (0.78–2.75)	0.230
Current smoking (n, %)	121 (27)	11 (25)	0.89 (0.44–1.81)	0.742
History of coronary artery disease (n, %)	37 (8)	13 (28)	4.42 (2.14–9.12)	<0.001
History of hypertension (n, %)	137 (30)	24 (52)	2.51 (1.36–4.62)	0.003
Diabetes mellitus (n, %)	52 (12)	7 (15)	1.38 (0.59–3.25)	0.460
Creatinine clearance (mean, SD)	124 (37)	119 (32)	1.00 (0.99–1.01)	0.363
Systolic blood pressure, mm Hg (mean, SD)	135 (18)	136 (16)	1.00 (0.99–1.02)	0.673
Body mass index (mean, SD)	28.8 (5.0)	29.2 (4.8)	1.02 (0.96–1.08)	0.590
Cholesterol ratio (mean, SD)	4.5 (2.6)	4.3 (1.3)	0.93 (0.74–1.15)	0.490
Left ventricular hypertrophy (n, %)	27 (6)	7 (15)	2.81 (1.15–6.87)	0.023
AHI (median, Q 1–3)	5.7 (1.5–16.9)	12.4 (6.9–40.6)	1.65 (1.30–2.09)	<0.001

*AHI* apnea-hypopnea index, *CI* confidence interval, *OR* odds ratio, *PVC* premature ventricular complexes, *Q* quartiles, *SD* standard deviation

**Table 2:** Demographic and clinical characteristics in patients with and without complex ventricular ectopy

	No complex *n* = 455	Any complex *n* = 47	OR (95% CI)	*P*
Age (mean, SD)	48 (11)	56 (9)	1.08 (1.05, 1.11)	<0.001
Male gender (n, %)	249 (55)	26 (55)	1.09 (0.66, 1.79)	0.752
Current smoking (n, %)	121 (27)	11 (23)	0.84 (0.47, 1.50)	0.552
History of coronary artery disease (n, %)	38 (8)	12 (26)	3.65 (1.91, 6.96)	<0.001
History of hypertension (n, %)	144 (32)	20 (43)	1.96 (1.18, 3.26)	0.009
Diabetes mellitus (n, %)	57 (13)	3 (6)	0.92 (0.42, 2.03)	0.838
Creatinine clearance (mean, SD)	125 (36)	118 (33)	0.99 (0.99, 1.00)	0.062
Systolic blood pressure, mm Hg (mean, SD)	134 (18)	145 (20)	1.02 (1.01, 1.04)	0.003
Body mass index (mean, SD)	29 (5)	30 (6)	1.01 (0.97, 1.07)	0.597
Cholesterol ratio (mean, SD)	4.5 (2.6)	4.3 (1.4)	0.94 (0.79, 1.12)	0.474
Left ventricular hypertrophy (n, %)	23 (5)	3 (6)	2.35 (0.95, 5.80)	0.065
AHI (median, Q 1–3)	5.9 (1.6, 17.1)	12.1 (6.0, 36.0)	1.42 (1.18, 1.72)	<0.001

*AHI* apnea-hypopnea index, *CI* confidence interval, *OR* odds ratio, *Q* quartiles, *SD* standard deviation

### Association between biomarkers and ventricular arrhythmias

**Fig. 3:**
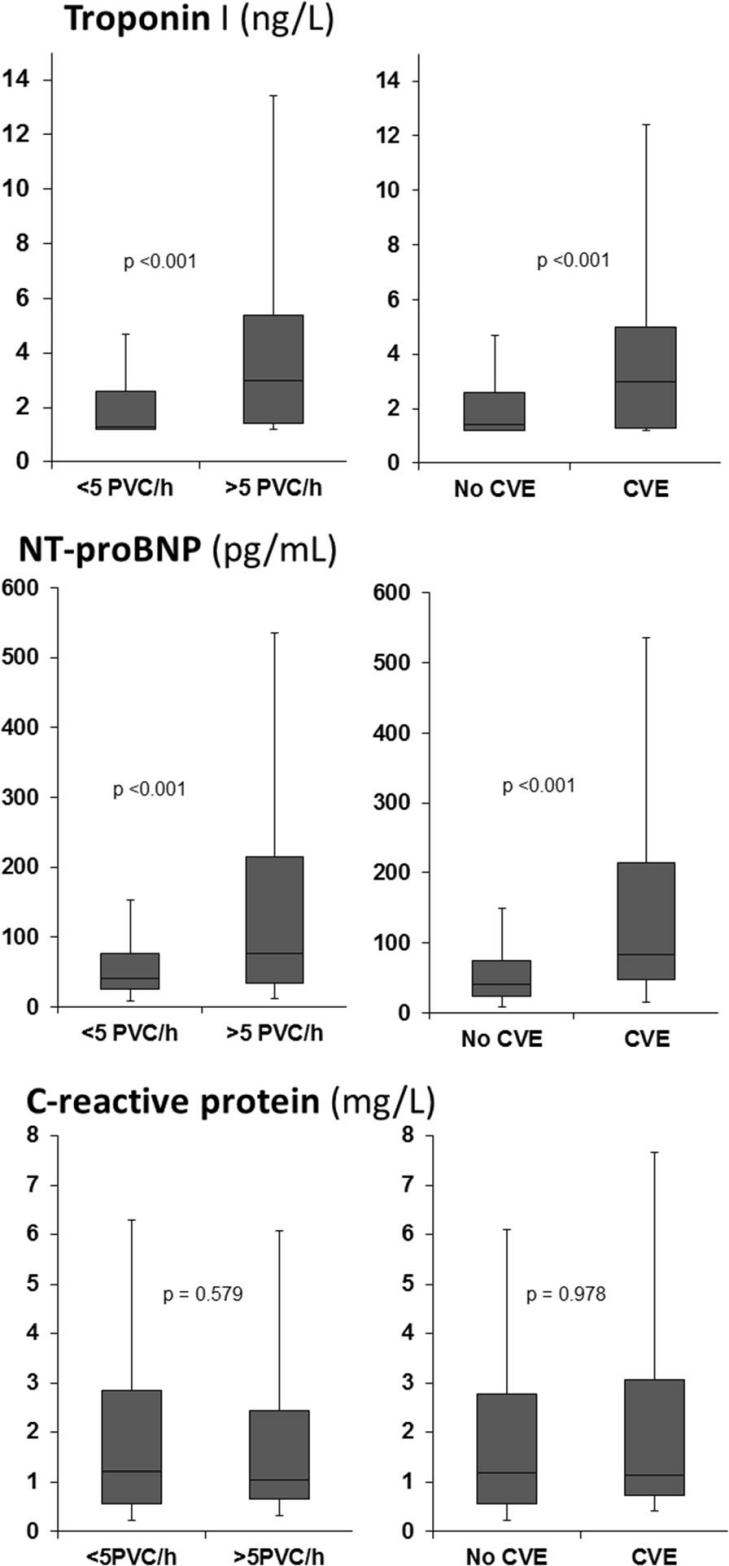
Serum-concentrations of biomarkers in 498 participants according to the presence or absence of frequent PVCs or complex ventricular ectopy

We found higher concentrations of hs-TnI (3.0 vs. 1.4 ng/L, *p* < 0.001) and NT-proBNP (75.7 vs. 41.3 pg/mL, *p* < 0.001) among the participants who had >5 PVC/h (Fig. 2). In contrast, hs-CRP concentrations did not differ between the two groups (1.0 vs. 1.2 mg/L, *p* = 0.978). The concentrations of hs-TnI (3.0 vs. 1.4 ng/L, *p* < 0.001) and NT-proBNP (82.6 vs. 41.0 pg/mL, *p* < 0.001) were also higher among participants with complex ventricular ectopy compared to those without, while we found no difference for hs-CRP concentrations (1.1 vs. 1.2 mg/L, *p* = 0.58) (Fig. 3).

Age, history of CHD, history of hypertension, left ventricular hypertrophy and AHI were associated with frequent PVC’s and included in the multivariate models (Table 3, Model 1). Each of the biomarkers hs-TnI (Model 2), NT-proBNP (Model 3) and hs-CRP (Model 4), was then added to the variables included in Model 1. The association between NT-proBNP and >5 PVC/h remained significant (OR 1.50 [95% CI 1.07–2.12], *p* = 0.020) in multivariate analyses, while the association between frequent PVC and hs-TnI was attenuated. NT-proBNP was also significantly associated with complex ventricular ectopy in analysis (OR 1.81 [1.27–2.57], *p* = 0.001) that adjusted for age, history of CHD, systolic blood pressure and AHI. The association between complex ventricular ectopy and hs-TnI was attenuated and no longer statistical significant in multivariate analyses (Table 4).

**Table 3:** Statistical significant determinants of >5 PVC/h, separate models for the 3 cardiovascular biomarkers. Multivariate logistic regression model, *n* = 498

	Model 1 OR (95% CI)	*P*	Model 2 OR (95% CI)	*P*	Model 3 OR (95% CI)	*P*	Model 4 OR (95% CI)	*P*
Age	1.07 (1.03–1.11)	0.001	1.06 (1.02–1.11)	0.005	1.05 (1.01–1.10)	0.014	1.07 (1.03–1.11)	0.001
History of coronary heart disease	2.80 (1.17–6.76)	0.021	2.52 (1.03–6.19)	0.044	2.00 (0.77–5.15)	0.152	2.76 (1.14–6.70)	0.025
History of hypertension	0.76 (0.34–1.66)	0.486	0.76 (0.34–1.67)	0.489	0.76 (0.34–1.68)	0.491	0.77 (0.45–1.70)	0.511
AHI (events/h)	1.41 (1.08–1.83)	0.010	1.37 (1.05–1.78)	0.019	1.46 (1.12–1.90)	0.006	1.42 (1.09–1.85)	0.010
Left ventricular hypertrophy	2.84 (1.07–7.57)	0.037	2.69 (1.00–7.26)	0.051	3.06 (1.13–8.32)	0.028	2.82 (1.06–7.53)	0.039
log hs-CRP (mg/L)							0.96 (0.69–1.33)	0.785
log NT-proBNP (pg/mL)					1.50 (1.07–2.12)	0.020		
log hs-TnI (ng/L)			1.32 (0.86–2.03)	0.205				

hs-CRP, NT-proBNP and hs-TnI were transformed by logarithm before regression analysis

*AHI* apnea-hypopnea index, *CI* confidence interval, *hsCRP* high-sensitive C-reactive protein, *hs-TnI* high-sensitive cardiac Troponin I, *NT-proBNP* N-terminal proB-type natriuretic peptide, *OR* odds ratio, *PVC* premature ventricular complexes

**Table 4:** Statistical significant determinants of complex ventricular ectopy, separate models for the 3 cardiovascular biomarkers. Multivariate logistic regression model, *n* = 498

	Model 1 OR (95% CI)	*P*	Model 2 OR (95% CI)	*P*	Model 3 OR (95% CI)	*P*	Model 4 OR (95% CI)	*P*
Age	1.06 (1.02–1.10)	0.003	1.06 (1.02–1.10)	0.007	1.04 (0.99–1.08)	0.091	1.06 (1.02–1.10)	0.003
History of coronary artery disease	2.48 (1.14–5.39)	0.022	2.29 (1.02–5.14)	0.044	1.49 (0.63–3.56)	0.365	2.50 (1.14–5.46)	0.022
Systolic blood pressure (mmHg)	1.02 (1.00–1.04)	0.033	1.02 (1.00–1.04)	0.057	1.02 (1.00–1.04)	0.037	1.02 (1.00–1.04)	0.034
AHI (events/h)	1.20 (0.95–1.51)	0.130	1.18 (0.93–1.50)	0.165	1.25 (0.98–1.58)	0.072	1.19 (0.94–1.51)	0.149
log hs-CRP (mg/L)							1.04 (0.76–1.44)	0.801
log NT-proBNP (pg/mL)					1.81 (1.27–2.57)	0.001		
log hs-TnI (ng/L)			1.18 (0.76–1.81)	0.467				

hs-CRP, NT-proBNP and hs-TnI were transformed by logarithm before regression analysis

*AHI* apnea-hypopnea index, *CI* confidence interval, *hs-CRP* high-sensitive C-reactive protein, *hs-TnI* high-sensitive cardiac Troponin I, *NT-proBNP* N-terminal proB-type natriuretic peptide, *OR* odds ratio

## Discussion

The main finding of the current study is that higher levels of NT-proBNP, but not hs-TnI or hs-CRP levels, are independently associated with the incidence of frequent ventricular ectopy and complex ventricular ectopy in a moderately large, community-based study population.

The present study assessed the relationship between two types of biomarkers for future CVD; low-level increments in circulating biomarkers and asymptomatic ventricular arrhythmias. We are aware of only one prior community-based study that reported higher unadjusted concentrations of NT-proBNP and CRP in persons with frequent PVC [17]. In that study, the combination of elevated CRP and frequent PVC increased the risk for future cardiovascular events, while NT-proBNP levels did not affect the association between frequent PVC and the outcomes. However, to the best of our knowledge, the associations between serum biomarkers and complex ventricular ectopy as well as the association between cardiac troponins and asymptomatic ventricular arrhythmias, have not previously been studied. We report that the concentration of NT-proBNP is associated with increased risk for both frequent PVCs and complex ventricular arrhythmias such as non-sustained ventricular tachycardias, bigeminy and trigeminy, independent of traditional risk factors for CVD. Regarding hs-TnI, the increase in serum concentration observed in participants with ventricular ectopy was not significant after adjustment for established hypertension, CHD, ECG-calculated left ventricular hypertrophy and AHI. In contrast to Sajadieh et al., we found no association between the inflammatory marker hs-CRP and any type of ventricular arrhythmia.

Different mechanisms may account for the association between NT-proBNP and ventricular arrhythmias. Left ventricular hypertrophy and mild left ventricular dysfunction are important determinants of low-level increments of both NT-proBNP and cardiac troponins [6], and are also associated with the risk of arrhythmias. Sub-clinical myocardial scarring and diffuse fibrosis may also cause both increased levels of cardiac markers as well as substrates for ventricular arrhythmias [24]. In contrast, subclinical myocardial inflammation, at least as reflected by hs-CRP concentrations, did not explain the tendency toward ventricular arrhythmias in our community-based cohort of relatively young subjects. The increase of circulating NT-proBNP could also be a result of the arrhythmia itself. However, as the observed arrhythmias were short-lasting and asymptomatic in this study, we find it unlikely that the arrhythmia itself has increased the NT-proBNP concentrations. Finally, recent data from the ASAP study suggest that the severity of OSA, as expressed by the AHI, could be independently associated with both asymptomatic arrhythmias [23] and troponin I [25]. At the time of preparation of the previous manuscript regarding frequent PVC’s, data concerning troponin I and NT-proBNP were not available, but the present data suggest that, at least for NT-proBNP, AHI is not the underlying mechanism.

A strength of this study is the community-based design and sample size. The generalizability is limited by the inclusion procedure, biasing participants with high weight, hypertension, snoring and daytime sleepiness. Thus, the risk factor profile current cohort is not reflective of the general population. Statistic procedures have been made to adjust for the presence of sleep apnea and CV risk factors. The lack of echocardiographic measures, particularly of left ventricular function, is another limitation of this study. Cardiac imaging data could potentially have added further information to the analysis; however, we included electrocardiographic signs of left ventricular hypertrophy in the study.

It is debated whether ventricular ectopy is an independent risk marker of mortality. In patients with established cardiac disease, the association may be explained by other established risk factors [26], while a recent report from a community-based study suggests that PVC’s predict HF and mortality [27]. The current study indicates that circulating biomarkers that assess low-level cardiac damage not detected by measures of ejection fraction ought to be included when investigating the prognostic influence by ventricular ectopy.

## Conclusions

In this study we demonstrate that the level of NT-proBNP is independently associated with frequent PVCs and complex ventricular ectopy, although none of the biomarkers examined are specific biomarkers of asymptomatic ventricular arrhythmias. We suggest that these relationships points to underlying mechanisms, not accounted for in traditional CV risk factors, which may contribute to the association between these biomarkers and CV death observed in the general population.

## Additional file

Additional file 1: Data set. (XLSX 60 kb)

## Abbreviations

AHI: Apnea-hypopnea index
ASAP: Akershus Sleep Apnea Project
BNP: B-type natriuretic peptide
CHD: Coronary heart disease
CI: Confidence intervals
CRP: C–reactive protein
CV: Cardiovascular
HF: Heart failure
hs: High sensitivity
hs-TnI: high sensitivity-troponin I
NT: N-terminal
NT-proBNP: N-terminal pro-B-type natriuretic peptide
OR: odds ratio
OSA: Obstructive sleep apnea
PVC: Premature ventricular complexes
Q: Quartiles
SD: Standard deviation
